# Identification of Cilia Genes That Affect Cell-Cycle Progression Using Whole-Genome Transcriptome Analysis in *Chlamydomonas reinhardtti*

**DOI:** 10.1534/g3.113.006338

**Published:** 2013-06-01

**Authors:** Alison J. Albee, Alan L. Kwan, Huawen Lin, David Granas, Gary D. Stormo, Susan K. Dutcher

**Affiliations:** * Department of Genetics, Washington University School of Medicine, St. Louis, Missouri 63110; † Center for Genomic Sciences and System Biology, Washington University, St. Louis, Missouri 63110

**Keywords:** flagella, deflagellation, ZMYND10, NXN, SPATA4, GLOD4

## Abstract

Cilia are microtubule based organelles that project from cells. Cilia are found on almost every cell type of the human body and numerous diseases, collectively termed ciliopathies, are associated with defects in cilia, including respiratory infections, male infertility, situs inversus, polycystic kidney disease, retinal degeneration, and Bardet-Biedl Syndrome. Here we show that Illumina-based whole-genome transcriptome analysis in the biflagellate green alga *Chlamydomonas reinhardtii* identifies 1850 genes up-regulated during ciliogenesis, 4392 genes down-regulated, and 4548 genes with no change in expression during ciliogenesis. We examined four genes up-regulated and not previously known to be involved with cilia (ZMYND10, NXN, GLOD4, SPATA4) by knockdown of the human orthologs in human retinal pigment epithelial cells (hTERT-RPE1) cells to ask whether they are involved in cilia-related processes that include cilia assembly, cilia length control, basal body/centriole numbers, and the distance between basal bodies/centrioles. All of the genes have cilia-related phenotypes and, surprisingly, our data show that knockdown of GLOD4 and SPATA4 also affects the cell cycle. These results demonstrate that whole-genome transcriptome analysis during ciliogenesis is a powerful tool to gain insight into the molecular mechanism by which centrosomes and cilia are assembled.

Cilia are hair-like organelles protruding from many types of cells in the human body. They play both signaling and mechanical roles in cells. At the base of cilia lie microtubule-based structures called basal bodies, which template and anchor cilia and recruit proteins needed for ciliary assembly. The presence of cilia and basal bodies is linked to the cell cycle in many organisms; mammalian cells that exit the cell cycle are quiescent, nonproliferating, and assemble cilia (Tucker *et al.* 1979). When cells re-enter the cell cycle, they absorb their cilia in late G1 (Rieder *et al.* 1979) and basal bodies convert to centrioles to become part of the spindle pole during mitosis. The prevailing hypothesis is that the cell cycle regulates the basal body/centriole and the assembly of cilia. Depletion of cdk or cyclin A or E eliminates centriole separation (Lacey *et al.* 1999). In many unicellular organisms such as Paramecium or Tetrahymena, cilia are retained during division.

In recent years, interest in cilia as an important organelle resurfaced due to a growing list of human diseases associated with ciliary defects, which cause a wide range of phenotypes that include renal cysts, liver disease, cognitive impairment, retinal degeneration, obesity, skeletal bone defects, laterality defects, and polydactyly (Albee and Dutcher 2012). Abnormal formation or function of these structures has been implicated as an underlying cause of many syndromes and disorders that have traditionally been recognized as disjoint conditions. The identification, characterization, and implication of human ciliopathy disease genes have greatly benefited from study in the model organism *Chlamydomonas reinhardtii* (Pazour *et al.* 2005). Chlamydomonas is a unicellular, green alga that has two cilia/flagella that are morphologically and biochemically similar to cilia found in humans. When environmental pH is lowered, Chlamydomonas cells shed their cilia and ciliogenesis begins immediately once a neutral pH is restored. The specific transcriptional induction of genes encoding many known cilia components during ciliogenesis have been widely reported and underscores one of the advantages of using Chlamydomonas as a model organism to study cilia and ciliogenesis.

Proteomic approaches using isolated Chlamydomonas cilia have generated an important list of ciliary components albeit with the caveat that low abundance and membrane proteins are not well represented (Pazour *et al.* 2005). This analysis has identified numerous structural components, but components that regulate cilia assembly or function such as those which preassemble dynein components in the cytoplasm were not identified (Mitchison *et al.* 2012). Genomic comparisons have also added to the list of ciliary components (Avidor-Reiss *et al.* 2004; Li *et al.* 2004; Merchant *et al.* 2007; Kwan *et al.* 2010; Hodges *et al.* 2011). These methods are complementary to proteomic methods, but they also generate an incomplete list because genes with conserved motifs such as kinases are discarded as a result of being in a nonciliated species. Many of the known ciliary components are up-regulated during ciliogenesis in Chlamydomonas. Previous methods to look at transcript levels focused on known genes (Lefebvre and Rosenbaum 1986), genes found by genomic comparisons (Li *et al.* 2004) or proteomics (Pazour *et al.* 2005), or used an incomplete version of the Chlamydomonas genome (Stolc *et al.* 2005). In addition, these studies focused on genes with increased fold change at 30 min and this single time point may also yield an incomplete list of ciliogenesis genes.

To generate a more complete picture of the genes required for ciliogenesis, we performed RNA sequencing (RNA-seq) (Nagalakshmi *et al.* 2008) and mapped the reads to the v5.3.1 Chlamydomonas genome assembly. We compared transcript abundance at 3, 10, 30, and 60 min during ciliogenesis with the predeflagellation transcript levels. We identified 1850 genes with an increased fold change of at least 2.5 at one or more of the time points. From this set, we analyzed four genes with homologs in humans using retinal pigment epithelial cells (hTERT-RPE1) expressing centrin-1/green fluorescent protein (GFP) and found that gene knockdown affects cilia, basal bodies/centrioles, and two genes also play an unexpected role in cell cycle progression.

## Materials and Methods

### Chlamydomonas sample preparation and RNAseq analysis

Chlamydomonas cell cultures were grown in 150 mL of Sager and Granick medium (R) to a concentration of 7.2 × 10^6^ cells per mL and 87.5% flagellated (Lux and Dutcher 1991). Cells were spun down in 50-mL conical tubes in a Sorvall RT6000 for 10 min at 2400 × g at room temperature, and resuspended in 25 mL of 10 mM HEPES buffer. A 5-mL aliquot was taken and diluted to 50 mL in R medium as “pretreatment” sample. Acetic acid (0.5N) was added dropwise to the remaining 20 mL with constant stirring to reach a pH of 4.1 as measured by a Corning pH meter 240 (Corning, NY) at 24°. After 45 sec, pH was restored to 7.1 with 0.5 N KOH. Deflagellation was confirmed by phase microscopy. Deflagellated cells were diluted 10-fold into R medium at 22°. Equal aliquots were taken at 0, 7, 27, and 57 min and spun in Sorvall RT6000 for 3 min at 2400 × g, bringing the total number of time points to five, labeled predeflagellation, 3, 10, 30, and 60 min.

RNA was extracted from Chlamydomonas cells with the QIAGEN RNeasy Mini Kit (QIAGEN, Valencia, CA). Yield of total RNA from each sample was ~5 µg. After DNase I treatment, 10 µg of total RNA was used to make cDNA library with Illumina RNA-Seq Prep kit (Illumina, San Diego, CA) and the cDNA libraries were sequenced on the Illumina Gene Sequencing Machine GAIIx.

The 36 base pair single end reads were aligned to the v5.3.1 Chlamydomonas genome using the TopHat alignment software suite (Trapnell *et al.* 2009). Transcript abundance for 18,757 gene models predicted on the v5.3.1 assembly were computed in FPKMs using the Cufflinks software suite (Trapnell *et al.* 2010).

### Expression profile clustering

The expression profile of each was determined for each gene from the ratio of its expression (FPKM) at each time point to the expression in the predeflagellation sample. A total of 1850 genes had ratios of at least 2.5-fold for at least one time point and were used to identify common profile types using the method of (Brady *et al.* 2007). To summarize, genes are first sorted by decreasing variance of the profile, and the top 75% of genes are then grouped by fuzzy k-means clustering. In contrast to standard k-means clustering, fuzzy k-means clustering assigns each profile a probability of membership in each cluster and allows multicluster membership for a given gene. Once initial membership is determined, the method determines the appropriate membership probability cut-off such that the average gene is assigned to one cluster (Brady *et al.* 2007). Similarity between clusters is then determined by the Pearson correlations between their mean profiles and a single-linkage hierarchical tree is generated. Branches of the tree with Pearson correlation >0.9 were merged into the final set of clusters and the mean profile for each cluster determined. Individual genes were then assigned to clusters for which the Pearson correlation between their profile and the mean profile of the cluster was >0.85, resulting in nearly every gene being assigned to exactly one cluster (Brady *et al.* 2007).

### Gene knockdown experiments

Multiple constructs expressing short hairpin (sh)RNA (Moffat *et al.* 2006) (Supporting Information, Table S1) were used for each gene and delivered into cells using the lentiviral system in the presence of 10 μg/mL polybrene. The medium containing the virus was incubated with hTERT-RPE1 cells expressing centrin-1/GFP (a kind gift from Dr. Alexey Khodjakov, Wadsworth Center) for 2−6 hr. The cells were allowed to recover and express the shRNA constructs overnight. The next day, 9 μg/mL puromycin was added to the medium to select for cells that had integrated and expressed the construct. Selection was maintained for 2 d. After 2 d, 1/5 of the cells were added to each of two wells of a six-well plate containing coverslips. RNA was isolated from the remaining cells and 1−3 μg of RNA was used to make cDNA using SuperScript III (Life Technologies, Carlsbad, CA). Knockdown levels were assessed by quantitative real time polymerase chain reaction (qRT-PCR) via an iCycler (Bio-Rad, Hercules, CA). Values were normalized using glyceraldehyde 3-phosphate dehydrogenase (GAPDH) as a control. Primers used for qRT-PCR analysis are listed in Table S2. The day after the cells were passed onto coverslips, 10 μM 5-ethynyl-2-deoxyuridine (EdU; Life Technologies) was added to one of the coverslips. Twenty-four hours after EdU was added, the cells were fixed and assessed for EdU uptake according to the manufacturer’s instructions. The medium was changed on the other coverslip to serum-free medium. The cells were maintained in serum-free medium for 72 hr before the cells were fixed with 4% paraformaldehyde and stained with an antibody against acetylated α-tubulin at 1:5000 (Sigma-Aldrich, St. Louis, MO) and DAPI to visualize the cilia and DNA, respectively.

### Plasmids

cDNA encoding GLOD4 (Accession BC008605), NXN (Accession BC009327), SPATA4 (Accession BC021731), and ZMYND10 (Accession BC033732) were ordered from Thermo Scientific. cDNAs were cloned in-frame with YFP in pFLRu-MCS-YFP (Feng *et al.* 2010) using PCR and In-Fusion HD cloning system (Clontech). For shRNAs that targeted the coding region, site-directed PCR was performed on the genes to mutate the targeted DNA sequence with synonymous changes. All genes were sequenced to ensure that they were in-frame with YFP and contained only the introduced synonymous mutations.

### Microscopy and data analysis

To assay EdU uptake, images were obtained with a PerkinElmer UltraVIEW VoX laser scanning disk confocal system equipped with a Zeiss Axio Observer Z1 microscope, α-Plan-Apochromat 40x/1.2 water or 63x/1.46 oil DIC M27 objectives, and EMCCD camera. Images were acquired with Volocity software. At least 100 nuclei (as judged by DAPI staining) were counted and the number of nuclei that also had EdU staining was recorded as the percent of EdU uptake. The control cells EdU uptake was set at 100% and the experimental samples were normalized to the controls.

To assay cilia and basal bodies, images were acquired with 63× or 40× objective (described previously). For percent ciliation, the number of cilia present on at least 100 cells was recorded. The controls were set to 100%, and the experimental samples were normalized to the controls. For cilia length, the length of at least 100 cilia (as judged by acetylated α-tubulin staining) was measured using Volocity software. For the number of basal bodies/centrioles per cell, the number of cells containing 2, 3, 4, 5, or ≥6 basal bodies/centrioles (as judged by centrin-1/GFP staining) were recorded in at least 100 cells and expressed as a percentage of the total number of basal bodies/centrioles. For the distance between the basal bodies/centrioles, the distance between mother and daughter basal bodies/centrioles (as judged by brightness of centrin-1/GFP) was measured using Volocity software.

## Results

### RNAseq generates a reliable transcriptome-wide ciliogenesis dataset

Illumina sequencing of mRNA isolated from predeflagellation, 3, 10, 30, and 60 min into ciliogenesis produced a total of 99.4 million 36-mer single-end reads, for an average of 19.9 million reads per time-point sample. This equates to 3.58Gb or a 32-fold coverage of the 112 Mb *Chlamydomonas* genome. TopHat (Trapnell *et al.* 2009) was used to compute expression levels of 18507 Augustus gene models. Expression values calculated by Cufflinks are reported in terms of fragments per kilobase transcribed per million reads mapped (FPKM) (Trapnell *et al.* 2010). In five independent sets of RNAseq sequencing (preshock, 3, 10, 30, and 60 min), 96% of RNAseq reads align to the v5.3.1 Chlamydomonas genome assembly. v5.3.1 is the most complete version of the Chlamydomonas genome and includes updated annotations, predictions of alternate transcripts, and incorporates gene expression data. Aligning our RNAseq data to the latest version of the genome gives a more accurate picture of up-regulated genes and includes transcripts not found in studies that used earlier versions of the genome (Pazour *et al.* 2005; Stolc *et al.* 2005). For genes to be categorized as expressed, we required that they have three FPKMs during at least one of our time points and we consider 10,813 genes to be expressed. Any gene with a time point to predeflagellation expression value ratio of 2.5 or greater is considered an up-regulated gene and we found 1850 predicted genes to be up-regulated at one or more time points (Table S3). We refer to these genes as up-regulated even though we recognize that these values are also a reflection of changes in transcription rate or message stability. There are 4392 genes down-regulated, and 4548 genes that do not alter their expression level during ciliogenesis (Table 1). The observation that the cell down-regulates almost half of its genes during ciliogenesis highlights the commitment of the cell to assemble cilia.

**Table 1:** Summary of RNAseq data

Predicted transcripts in the genome	19,529
Genes aligned to genome	18,757
Genes with FPKM ≥3	10,790
Genes with a decreased fold change ≥2.5-fold	4392
Genes without a change in level	4548
Genes with increased fold change of ≥2.5-fold	1850

To evaluate the reliability of our data, we compared it with previous methods of identifying ciliogenesis genes. The flagellar proteome contains 624 genes with v5.3.1 Chlamydomonas gene predictions with two or more peptides (Pazour *et al.* 2005). Of these, 357 are found in our RNAseq dataset (Table 2). Of the 1429 genes in the flagellar proteome identified by a single peptide that have v5.3.1 gene predictions, 483 are found in our dataset. This finding suggests that not all structural proteins in the flagella show up-regulation. Stolc *et al.* (2005) used microarray analysis to identify genes up-regulated 30 min after deflagellation, similar to our method. Of the 214 genes with v5.3.1 gene matches that they identified, 196 genes are in our RNAseq dataset (Table 2). Of the 18 genes identified by Stolc *et al.* not found in our RNAseq data, 10 are heat shock proteins and may represent a stress response rather than a ciliogenesis response. Five of genes have either no similarity to known genes or no predicted domains. The remaining three genes include a DEAD-box helicase, a potential splicing factor, and a membrane transporter. RNAseq of mouse tracheal epithelial cells (MTECs) undergoing differentiation to become multiciliated, identified 649 genes (Hoh *et al.* 2012) and of these 397 have homologs in Chlamydomonas. There are 222 genes (56%) with RNAseq support in both Chlamydomonas and mouse. The MTECs generate 200−300 cilia per cell and must duplicate their basal bodies in addition to generating cilia. Some of the genes identified in MTECs may be due to basal body duplication or multiciliarity and may explain why only roughly half of those genes are found in our data. This comparison shows that overall our data agrees with previously published studies and reports many new genes that are likely to be involved in ciliogenesis.

**Table 2:** Comparison with previous published results

Evidence	No. Genes Supported by RNAseq	No. Genes with v5 Predictions
Flagellar proteome 2 or more peptides*^a^*	357	624
Flagellar proteome 1 or more peptides*^a^*	483	1429
Microarray *^b^*	196	214
RNAseq mouse tracheal cells ^c^	222	397

a Pazour *et al.* 2005.

b Stolc *et al.* 2005.

c Hoh *et al.* 2012.

**Figure 1:**
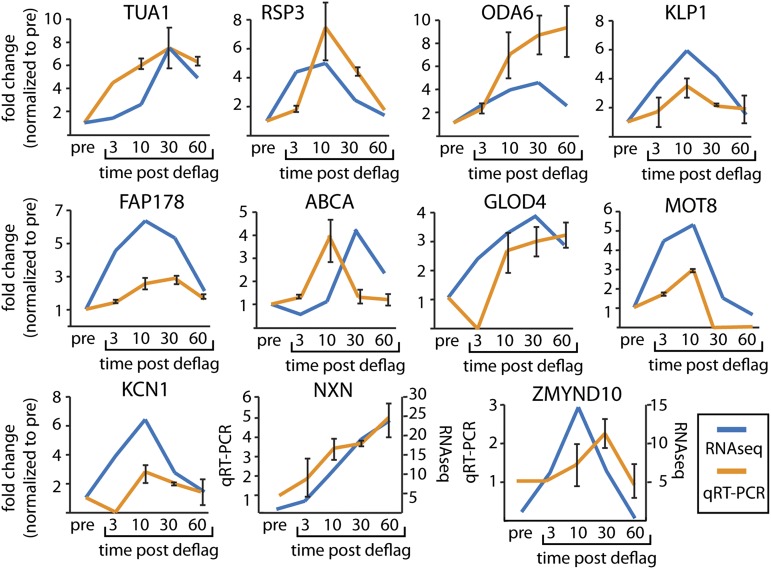
Expression profiles during ciliogenesis are similar between RNAseq and qRT-PCR. Genes with previous support for involvement in ciliogenesis (TUA1, RSP3, ODA6, KLP1, and FAP178) and new genes (ABCA, GLOD4, MOT8, KCN1, NXN, and ZMYND10) were examined for the up-regulation pattern by RNAseq and qRT-PCR. Error bars on qRTPCR data are the average of two independent experiments. Expression profiles generally agree between the two methods.

We further tested for genes up-regulated in the RNAseq experiment by using qRT-PCR of five genes with previous support [α-tubulin (*TUA1*), radial spoke protein (*RSP3*), outer dynein arm (*ODA6*), kinesin (*KLP1*), and flagellar protein 178 (*FAP178*)] and found agreement between our qRT-PCR and RNAseq data (Figure 1). We also examined six genes with no previous ciliary support [ABC transporter (*ABCA*), glyoxylase domain containing protein (*GLOD4*), lipid phosphatase (*MOT8*), potassium voltage gated channel (*KCN1*), nucleoredoxin (*NXN*), and MYND domain containing protein (*ZMYND10*)]. The human gene names are given for ABCA, GLOD4, NXN, and ZMYND10 because they do not have Chlamydomonas gene names. All other gene names refer to the Chlamydomonas gene names. We find general agreement of up-regulation between RNAseq and qRT-PCR (Figure 1). Some of the differences may be attributable to the different methods of normalization between RNAseq and qRT-PCR. RNAseq is normalized against transcript levels across the transcriptome and qRT-PCR is normalized against a single control gene (GAPDH). Absolute differences between RNAseq and qRT-PCR may reflect changes in a single gene verses changes in the entire transcriptome.

### Gene expression analysis reveals early ciliogenesis-regulation programs

**Figure 2:**
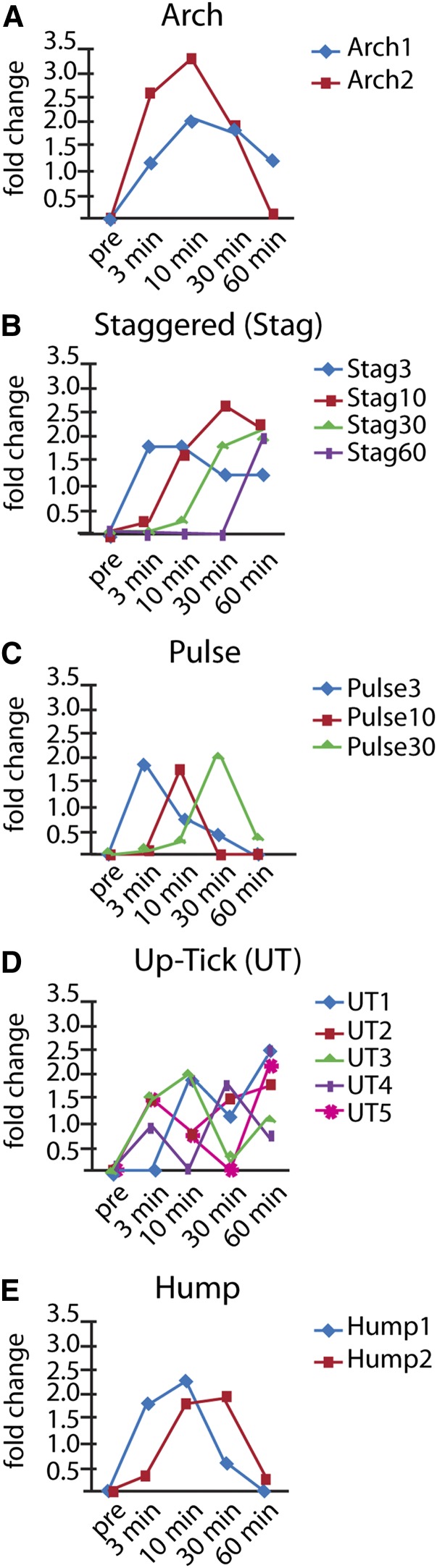
Profile clustering identifies 16 principal up-regulation expression profiles organized into five pattern groups. (A) The Arch pattern shows increased expression at 3 min, a peak expression at 10 min, and then decreasing expression at 30 and 60 min. This profile pattern group accounts for 37% of up-regulated gene profiles. The first and second most common principal expression profiles are found with this pattern: Arch1 (n = 336; 18%) and Arch 2 (n = 343; 19%), respectively. The Arch1 pattern shows expression that peaks at 10 or 30 min but is still up at 60 min. The Arch2 pattern is similar to Arch1 except that the genes are not up-regulated at the 60-min time point. (B) The Staggered (Stag) pattern shows genes with a burst of expression at the 3, 10, 30, or 60 min time points. The third, fourth, fifth, and tenth most common expression profiles are found with this pattern: Stag30 (n = 226; 12%), Stag10 (n = 189; 10%), Stag60 (n = 165; 9%), and Stag3 (n = 49; 3%). (C) The Pulse pattern shows up-regulation at a single time point and accounts for 11% (n = 196) of the up-regulated genes. (D) The Up-Tick pattern describes genes that show increased expression at one time point followed by down-regulation at another time point followed by up-regulation at a third time point. Up-Tick patterns comprise 3% (n = 51) of up-regulated genes and can be further subdivided by the time point of the up-tick. (E) Hump patterns comprise 16% (n = 290) and are profiles that are pulse-like, but significant up-regulation is sustained over two consecutive time points. Of the remaining fraction, 0.5% (n = 9) show profiles that are outliers in that their profiles are not adequately similar to any principal expression profile found in this analysis.

We examined the expression patterns during our time course to determine a set of principal regulation profiles for genes that are up-regulated during the first 60 min of cilia regeneration in Chlamydomonas. We chose these to look at early timepoints based on previous studies showing that many flagellar mRNAs begin to increase between 10 and 30 min and return to predeflagellation levels 2−3 hr postdeflagellation (Silflow and Rosenbaum 1981; Schloss *et al.* 1984). Sixteen principal regulation profiles are identified and then grouped into five main patterns (Figure 2). The Arch group, containing patterns Arch1 and Arch2, shows increased expression at 3 min, a peak expression at 10 min, and then decreasing expression at 30 and 60 min (Figure 2A). Arch2 is the most common pattern with 343 genes, whereas Arch1 is the second most common pattern with 336 genes (Table S3). Altogether, 37% of genes display the Arch pattern. The Staggered (Stag) group consists of four profile patterns that show a burst of expression at the 3, 10, 30, or 60 min time points with continued expression thereafter (Figure 2B). Stag30 is the third most common pattern with 226 genes, Stag10 is the fourth most with 189 genes, and Stag60 and Stag3 are the fifth and tenth most common with 165 and 49 genes, respectively. Together the six patterns in the Arch and Staggered groups contain 71% of the up-regulated genes. The remaining up-regulated genes are categorized into three groups of profile patterns: Hump, Pulse, and Up-Tick (Figure 2, C−E; the number of genes in each pattern is listed in Table S3). The Hump pattern shows significant up-regulation over the course of two time points, either at 3 min and 10 min (Hump1) or 10 min and 30 min (Hump2; Figure 2C). The Pulse pattern is similar to Hump patterns, but significant up-regulation is sustained for a single time point (Figure 2D). We observe a pulse pattern at each of the 3-minute (Pulse3), 10-minute (Pulse10) or 30-minute (Pulse30*)* time points. We note that a fraction of up-regulated genes categorized as Stag60 may actually exhibit a 60-minute pulse if further data were gathered at later time points. Up-Tick patterns have more complicated profiles with multiple peaks and can be further subdivided by the time points of their expression peaks (Figure 2D).

We then examined structural ciliogenesis genes to determine whether these genes may show similar patterns of expression. Of the IFT genes and associated motors, 90% of the genes fell into the Arch category and 10% of the remaining genes are in the Stag group (Table S4), suggesting that most genes involved with IFT display an Arch pattern of expression. Axonemal dyneins display more varied expression patterns but still show an increase of genes with an Arch expression pattern, with 60% of genes in Arch, 27% in Stag, 8% in Pulse, and 5% in Hump (Table S4). Radial spoke genes and central pair genes also show an increase in Arch expression pattern over all genes identified at 78% and 84%, respectively (Table S4). Overall, structural ciliogenesis genes are more likely to have Arch expression patterns.

### RNAseq identifies genes involved in ciliogenesis

**Figure 3:**
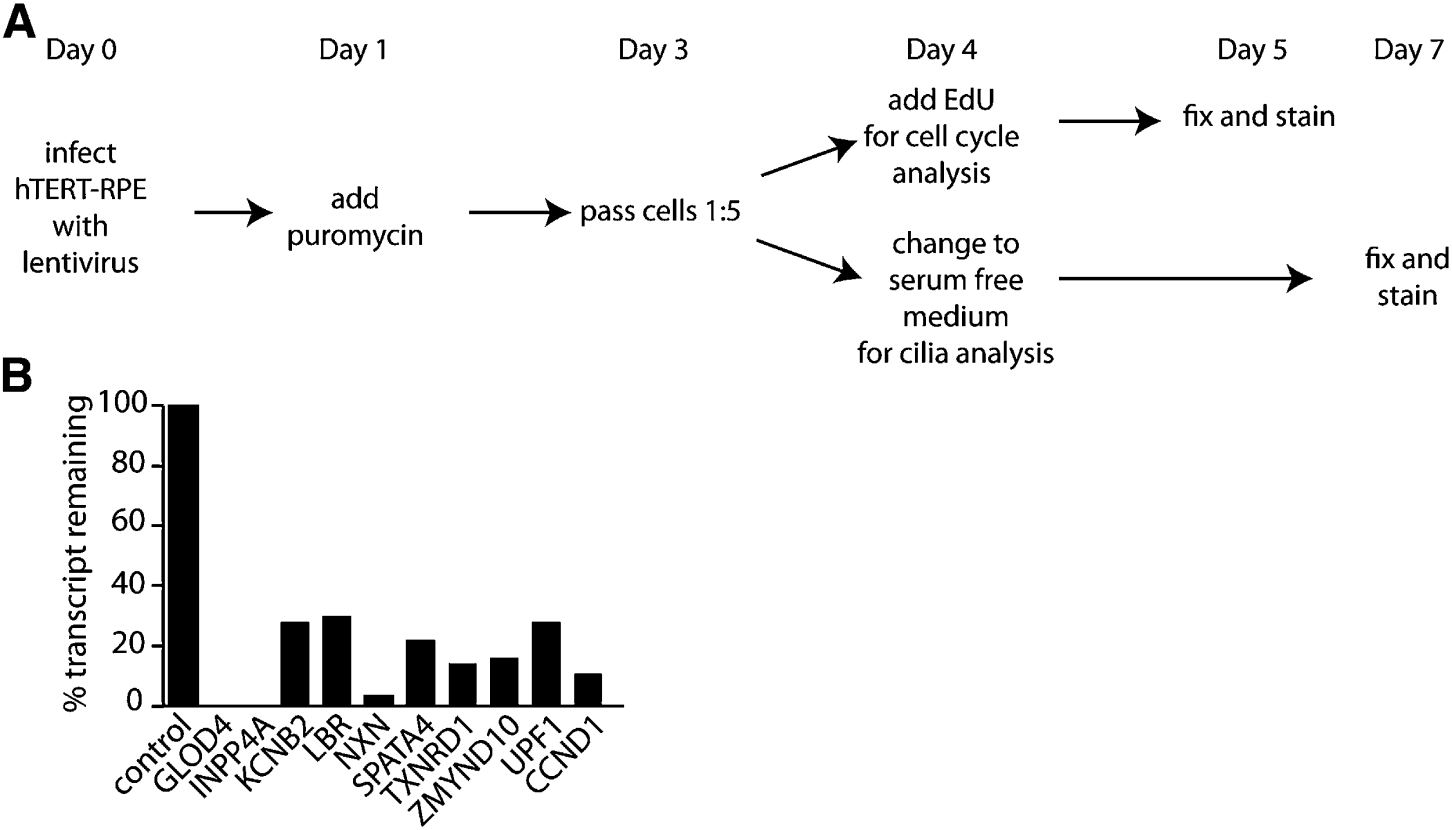
Knockdown by shRNA as a method to validate our dataset. (A) On Day 0, hTERT-RPE1 expressing centrin/GFP was infected with lentivirus containing shRNAs. The next day, puromycin was added to select for cells that incorporated the shRNAs. After selection (Day 3), cells were passed 1:5 and plated onto coverslips. On Day 4, one set of coverslips received EdU and the other set had their medium changed to serum-free to induce cilia growth. After 24 hr in EdU (Day 5), the coverslips were fixed and stained. After 3 d in serum-free medium (Day 7), the coverslips were fixed and stained for acetylated α-tubulin. (B) The amount of transcript remaining for each gene normalized to GAPDH. Although we used 4−5 shRNAs per gene, only the construct with the greatest degree of knockdown is shown here.

We chose four genes without previous associations with ciliogenesis that were up-regulated by RNAseq in Chlamydomonas and also had human homologs (Table 3) for further analysis in immortalized human retinal pigment epithelial cells (hTERT-RPE1) expressing centrin-1/GFP. The RPE cells assemble a primary cilium upon serum starvation. Each gene was knocked-down by lentiviral shRNAs and selected with puromycin to enrich for cell populations that received the shRNAs (Figure 3A). Knockdown levels were accessed by qRT-PCR (Figure 3B). For the control cells, a lentiviral shRNA targeted against a scrambled construct was used. After establishing that our genes of interest showed knock down with transcript reduced to 30% or less (Figure 3B), we examined five phenotypes: the percent of cells with a cilium, cilia length, numbers of basal bodies/centrioles, distance between basal bodies/centrioles, and cell-cycle defects as determined by EdU staining.

**Table 3:** Summary of genes in this study

			FPKM Values (Transcript Level)					Fold Change			
Chlamydomonas	*H. sapiens*	Description	Pre	3 Min	10 Min	30 Min	60 Min	3 Min	10 Min	30 Min	60 Min
Cre17.g704350	GLOD4	Glyoxalase domain	3.55	8.10	12.50	15.05	11.58	2.28	3.52	4.24	3.26
g1771	NXN	Nucleoredoxin	4.22	16.29	88.76	190.26	246.42	3.86	21.03	45.07	58.38
FAP178	SPATA4	Unknown function	11.99	94.49	163.70	111.98	28.03	7.88	13.65	9.34	2.34
Cre08.g358750	ZMYND10	Zinc finger and MYND domains	4.08	36.79	117.92	34.19	0.71	9.02	28.92	8.38	0.17

### Cilia require ZMYND10

**Figure 4:**
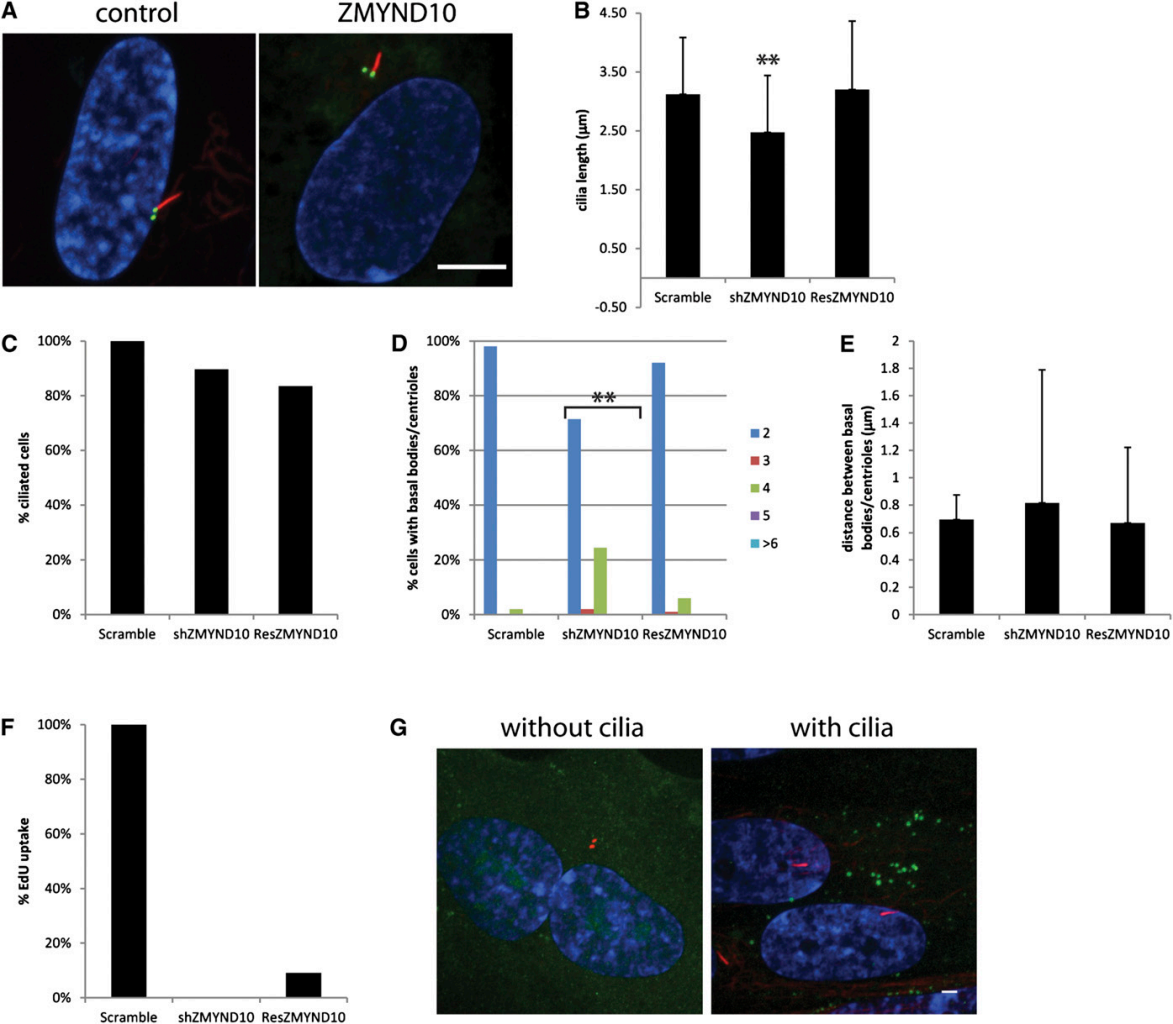
Depletion ZMYND10 causes short cilia. (A) Micrograph of control cells showing normal cilia (left) and a micrograph of a representative image from ZMYND10 knockdown. Scale bar is 10 μm. Blue, DNA; red, acetylated α-tubulin; green, centrin/GFP. (B) Graph showing cilia length after gene knockdown. Error bars represent SD from at least 100 cilia per gene knockdown. Significance was determined using a Student’s *t*-test with two tails and unequal variance. **P < 0.001. (C) Percent ciliated cells normalized to the control. (D) Percent of cells with 2, 3, 4, 5, or ≥6 basal bodies/centrioles. Bracket indicates gene knockdowns that have increased numbers of basal bodies/centrioles compared with the control. (E) Distance between the mother and daughter basal bodies/centrioles. Error bars represent SD from at least 100 cells per gene knockdown. Significance was determined using a Student’s *t*-test with two tails and unequal variance. (F) Percent of cells that up took EdU normalized to the control. (G) Examples of localization of ZMYND10 in cells with and without cilia. Green, gene-YFP; red, centrioles left panel, cilia right panel; blue, DNA.

ZMYND10 is a zinc finger protein with an MYND motif. The MYND domain is commonly recognized as being required for protein−protein interactions. ZMYND10 is also one of several loss of function genes associated with several types of cancers, but its role is unclear (Tischoff *et al.* 2005; Yi Lo *et al.* 2006; Lai *et al.* 2007; Riquelme *et al.* 2007; Lorente *et al.* 2009). Although not previously shown to be involved in ciliogenesis, it is found only in organisms with cilia and was up-regulated almost 29-fold at the 10 min time point in our dataset. This gene was also found to be more than 4-fold up-regulated in ciliated epithelial cells (Hoh *et al.* 2012). Knockdown of ZMYND10 caused short cilia (Figure 4, A and B) without affecting the number of cells with cilia (Figure 4C). There are also increased numbers of basal bodies/centrioles when ZMYND10 levels were reduced (Figure 4D) without affecting the distance between them (Figure 4E). Cilia length and basal body/centriole number are rescued by a shRNA resistant version of ZMYND10 (Figure 4, B and D) demonstrating that these phenotypes are the result of ZMYND10 knockdown and not to the result of off-target effects. Knockdown of ZMYND10 reduces the number of cells incorporating EdU, although this phenotype is not rescued by the shRNA resistant ZMYND10, indicating this phenotype is not directly related to ZMYND10 knockdown (Figure 4F). The rescue construct of ZMYND10 contains a C-terminal YFP tagged so we could examine its localization. We find that ZMYND10 localizes to puncta in the cytoplasm when cilia are present or not (Figure 4G). These puncta might be part of the secretory system such as vesicles budding from the Golgi as has been shown for other proteins with ciliary phenotypes (Follit *et al.* 2006; Knodler *et al.* 2010; Donaldson and Jackson 2011). Alternatively, they may be part of a preassembly complex for axonemal components (Mitchison *et al.* 2012).

### NXN is required for ciliogenesis

**Figure 5:**
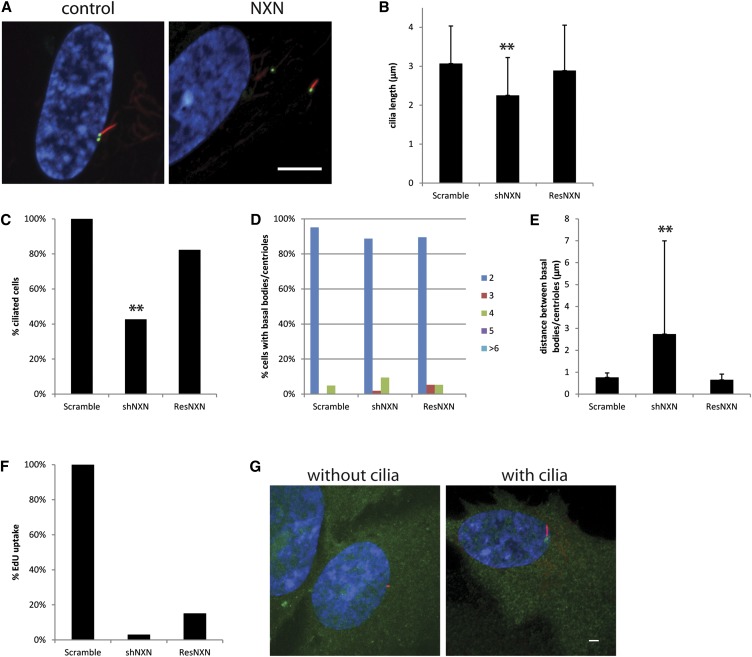
NXN knockdown causes cells to have short cilia and basal bodies/centrioles to be far apart. (A) A representative image from NXN knockdown. Scale bar is 10 μm. Blue, DNA; red, acetylated α-tubulin; green, centrin/GFP. (B) Cilia length after gene knockdown. Error bars represent SD from at least 100 cilia per gene knockdown. Significance was determined using a Student’s *t*-test with two tails and unequal variance. **P < 0.001. (C) Percent ciliated cells normalized to the control. **Fewer cilia. (D) Percent of cells with 2, 3, 4, 5, or ≥6 basal bodies/centrioles. (E) Distance between the mother and daughter basal bodies/centrioles. Error bars represent SD from at least 100 cells per gene knockdown. Significance was determined using a Student’s *t*-test with two tails and unequal variance, **P < 0.001. (F) Percent of cells that incorporated EdU normalized to the control. (G) Examples of localization of NXN in cells with and without cilia. Green, gene-YFP; red, centrioles left panel, cilia right panel; blue, DNA.

Thioredoxins are general disulfide oxidoreductases that alter protein structure by reducing disulfide bonds. Nucleoredoxin (NXN) is a thioredoxin. NXN functions in the Wnt signaling pathway to regulate protein levels of Disheveled (Funato *et al.* 2010). Although the connection between cilia and Wnt signaling has been controversial (May-Simera and Kelley 2012), we find NXN to be up-regulated 58-fold in our data and examined its knockdown phenotype. Knockdown of NXN causes fewer cells to have cilia (Figure 5, A and C), and the cells that do have cilia, display short cilia (Figure 5B). Although knockdown of NXN did not alter the numbers of basal bodies/centrioles (Figure 5D), the basal bodies/centrioles were farther apart than in control cells (Figure 5E). Fewer cells with NXN knockdown take up EdU although this phenotype was not rescued by a shRNA resistant NXN construct (Figure 5F) indicating that cell cycle progression is not the result of reduced NXN levels. The ciliary phenotypes, length, percent of cells with cilia, and basal body/centriole distance are rescued by the shRNA resistant NXN construct (Figure 5, B, C, and E), indicating that these phenotypes are directly related to reduced levels of NXN. YFP-tagged NXN localize to both the cytoplasm and nucleus in RPE cells as shown previously for COS-7 (Kurooka *et al.* 1997) and NIH3T3 cells (Funato *et al.* 2006).

### Ciliogenesis requires GLOD4

**Figure 6:**
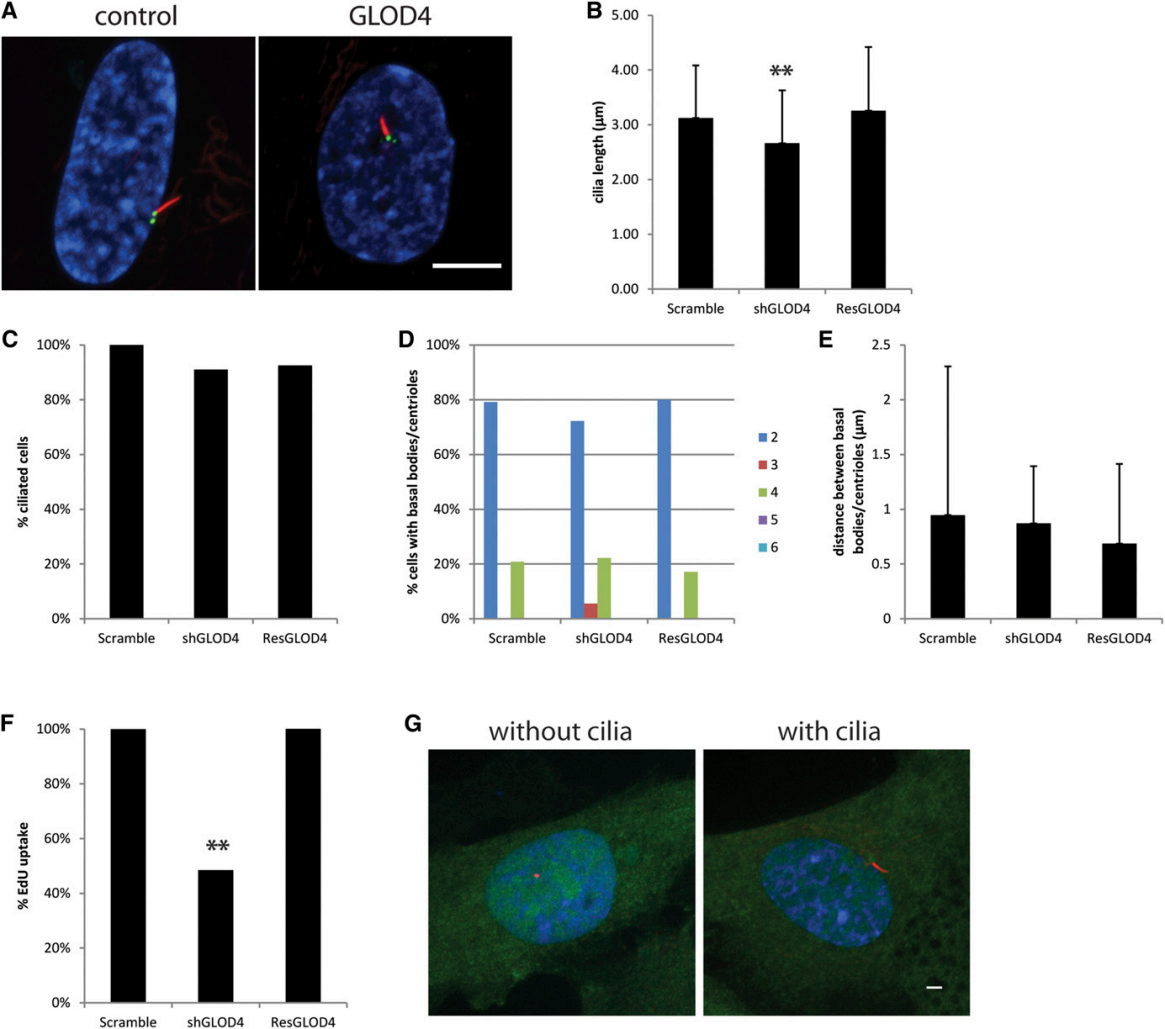
GLOD4 causes short cilia and altered cell-cycle progression. (A) A representative image from GLOD4 knockdown. Scale bar is 10 μm. Blue, DNA; red, acetylated α-tubulin; green, centrin/GFP. (B) Cilia length after gene knockdown. Error bars represent SD from at least 100 cilia per gene knockdown. Significance was determined using a Student’s *t*-test with two tails and unequal variance. **P < 0.001 (C) Percent ciliated cells normalized to the control. (D) Percent of cells with 2, 3, 4, 5, or ≥6 basal bodies/centrioles. (E) Distance between the mother and daughter basal bodies/centrioles. Error bars represent SD from at least 100 cells per gene knockdown. (F) Percent of cells that incorporated EdU normalized to the control. (G) Examples of localization of GLOD4 in cells with and without cilia. Green, gene-YFP; red, centrioles left panel, cilia right panel; blue, DNA.

GLOD4 is a glyoxalase protein that is part of the enzymatic detoxification system in mitochondria (Mannervik 2008). We chose to look at GLOD4 because it had no previous support for a role in cilia, although it was shown to be differentially up-regulated in a canine model of retinal degeneration (Genini *et al.* 2010), a common ciliopathy phenotype. Knockdown of GLOD4 did not alter the number of cells with cilia; however, the cilia were shorter than the control (2.7 μm ± 1.0 μm *vs.* 3.1 μm ± 1.0 μm; Figure 6, A and B). The short cilia phenotype is rescued by a shRNA resistant copy of GLOD4 showing that the short cilia phenotype is because of reduced levels of GLOD4 and not from off target effects (Figure 6B). The percent of cells with cilia, the numbers of basal bodies/centrioles and the distance between basal bodies/centrioles is not altered when GLOD4 levels are reduced (Figure 4, C−E). GLOD4 knockdown cells take up less EdU, indicating there is a cell-cycle progression defect that can be rescued by a shRNA-resistant GLOD4 (Figure 6F). GLOD4-YFP localized ubiquitously throughout the cell and did not specifically localize to the basal bodies/centrioles or cilia (Figure 6G).

### Knockdown of SPATA4 causes few cells to build a cilium

**Figure 7:**
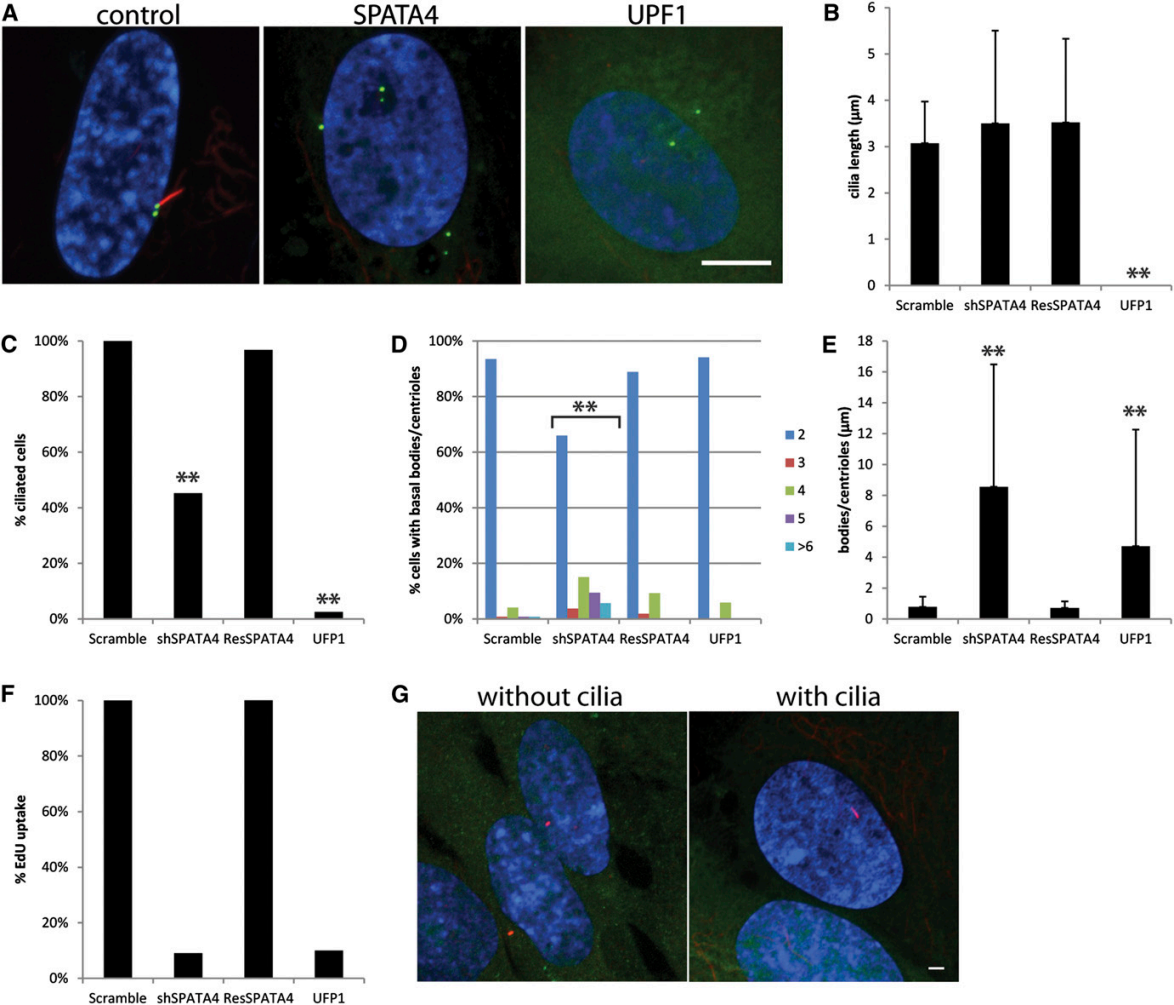
SPATA4 knockdown arrests cells in S-phase. (A) A representative image from SPATA4 and UPF1 knockdown. Scale bar is 10 mm. Blue, DNA; red, acetylated a-tubulin; green, centrin/GFP. (B) Graph showing the percent ciliated cells normalized to the control. **Because there were no cilia after UPF1 knockdown, cilia lengths could not be measured. (C) Percent ciliated cells normalized to the control. **Fewer cilia. (D) Percent of cells with 2, 3, 4, 5, or >6 basal bodies/centrioles. Bracket indicates gene knockdowns that have increased numbers of basal bodies/centrioles compared to the control. (E) Distance between the mother and daughter basal bodies/centrioles. Error bars represent SD from at least 100 cells per gene knockdown. Significance was determined using a Student's t-test with two tails and unequal variance. **P <0.001. (F) Percent of cells that incorporated EdU normalized to the control. (G) Localization of SPATA4 in cells with and without cilia. Green, SPATA4-YFP; red, centrioles left panel, cilia right panel; blue, DNA.

SPATA4 (FAP178 in Chlamydomonas) is found in the flagellar proteome (Pazour *et al.* 2005) and was up-regulated more than 13-fold by RNAseq (this study). SPATA4 is also a good candidate for having a cilia-related phenotype because of its high expression in the testes of many animals (Liu *et al.* 2004a,b; Liu *et al.* 2005a). In our assay, cells that had reduced mRNA levels of SPATA4 have no change in the length of cilia (Figure 7, A and B); however, fewer cells had a cilium (Figure 7C). Few SPATA4 knockdown cells incorporate EdU (Figure 7F), which suggests a cell-cycle arrest. Surprisingly, cells have increased numbers of basal bodies/centrioles that are at least 8 μm apart (Figure 7, D and E), whereas the control basal bodies are never more than 0.76 ± 0.08 μm apart. All of these phenotypes could be rescued by a shRNA-resistant copy of SPATA4 (Figure 7, B−F). SPATA4 localized to the cytoplasm and nucleus but did not specifically localize to either the centrioles or cilia (Figure 7G). This finding is consistent with cytoplasmic and nuclear localization of SPATA4 observed in HEK293T (Duarte *et al.* 2011). Similar to the phenotypes observed after SPATA4 knockdown, depletion of the origin of replication complex 2 leads to S-phase arrest with excess centrioles (Prasanth *et al.* 2004). To ask whether S-phase arrest always causes cilia and basal body/centriole defects, we looked at the ciliogenesis phenotypes from UPF1 knockdown, a gene known to arrest cells in early in S-phase (Azzalin and Lingner 2006). It shows very similar phenotypes to SPATA4. Few cells assemble cilia and few cells uptake EdU (Figure 7, A, B, and E). The basal bodies/centrioles are also far apart (Figure 7D); however, unlike SPATA4 knockdown, there were normal numbers of basal bodies/centrioles (Figure 7C). Thus S-phase arrest does not always lead to overduplication of basal bodies/centrioles (Azimzadeh *et al.* 2009; Loncarek *et al.* 2010; Steere *et al.* 2011). SPATA4 may have an important role in inhibiting basal body duplication.

## Discussion

We used whole transcriptome analysis to identify 1850 genes up-regulated at least 2.5-fold during ciliogenesis in Chlamydomonas. Many genes required for ciliogenesis are up-regulated, including all 16 IFT Complex B genes, six IFT Complex A genes, three anterograde motor and associated proteins, and four retrograde motor and associated proteins (Table S4). All 41 proteins, all 18 radial spoke proteins, and 13 of 15 central pair proteins had their transcripts up-regulated (Table S4). Additional genes that have been implicated in cilia assembly and function are found in our RNAseq data. These include MORN1 (FAP266) that has been implicated in basal body assembly in Toxoplasma (Lorestani *et al.* 2010), PIH1D1, which is part of the prefoldin complex and interacting proteins Reptin and Pontin have been implicated in cystic kidney disease in zebrafish (Sun *et al.* 2004) and in Chlamydomonas. Tubby is linked to adult onset obesity (Coleman and Eicher 1990) and retinal degeneration (Ohlemiller *et al.* 1995) in mice and the Tubby like proteins (TULP1-4) are also implicated or directly involved in cilia related processes (Mukhopadhyay and Jackson 2011). We find evidence of up-regulation for Chlamydomonas genes with human homologs that were previously predicted to be involved in cilia by comparative genomics studies, but for which there existed neither qRT-PCR nor proteomic evidence. Our data identify human genes with Chlamydomonas homologs that are up-regulated during ciliogenesis. This set includes six genes that have existing evidence of their involvement in cilia or ciliogenesis (KAT, KIF21A, TXNDC3, DYNLT1, TUBB, ACT). There are other genes with existing nonciliopathy disease genes or mutant phenotypes to which our data assign novel cilia annotations, thereby suggesting some cilia involvement in new diseases or mutant phenotypes, including COACH syndrome, Noonan syndrome, cleft lip and palate gene, polycystic ovary syndrome, and perinatal lethality in mice. The largest category in the remainder of the set contains solute carriers/transporters, followed by Golgi/membrane trafficking proteins, chaperonins, and kinases and phosphatases. The remainder of the protein annotation groups includes proteins that are involved in or that are associated with mitochondria, lipid and inositol metabolism, cell cycle, ubiquitin, proteins that act on disulfide bonds, and DNA repair. These results suggest new areas in which there may be cilia involvement and indicate the potential of using Chlamydomonas as a model organism for the study of these diseases and phenotypes.

We observed the LBR gene to be up-regulated in our dataset. In Chlamydomonas, the LBR homolog is involved in sterol biosynthesis. Sterols play crucial roles in membranes where they regulate membrane permeability and fluidity by interactions with other lipids and proteins. Sterols are frequently enriched in detergent-resistant membranes, which organize molecules involved in specialized signaling processes that include Agg2 and Agg3 in Chlamydomonas. Chlamydomonas synthesizes ergosterol rather than cholesterol as in animal cells. We looked at other genes in the ergosterol pathway to ask if they are up-regulated after deflagellation. This pathway uses the mevalonate-independent 2-C-methyl-D-erythritol 4-phosphate pathway to synthesize delta-3-isopentenyl-pyrophosphate as the start of the isoprenoid pathway. Chlamydomonas has homologs of 11 of the 12 genes involved in ergosterol biosynthesis in yeast. It may have additional genes that are needed for a sterol C24- methylation-C25-reduction (Δ(25(27))-olefin) step not observed in yeast (Miller *et al.* 2012). None of the genes in the mevalonate-independent 2-C-methyl-D-erythritol 4-phosphate pathway are up-regulated, whereas 9 of the 11 genes in the IPP pathway are up-regulated more than 2-fold (Table S5). These results implicate sterols as being important for ciliogenesis.

We analyzed the function of four human homologs and found knockdown of all four genes cause ciliogenesis defects in human retinal pigmented epithelial cells, which indicates that up-regulation during ciliogenesis in Chlamydomonas is an excellent way to identify genes that are conserved in ciliogenesis. To our surprise, knockdown of two genes in RPE cells also had profound effects on the cell cycle.

We expected to find genes that produced cells with few cilia or short cilia. This is a common phenotype seen with other genes with ciliary functions including the IFTs, kinesins and dyneins. As we expected, knockdown of ZMYND10, NXN, and GLOD4 affected cilia formation and showed short cilia. Previous work suggests a wide range of functions for these genes. The expression of ZMYND10 is down-regulated in cancers and may be a tumor suppressor (Liu *et al.* 2003; Shao *et al.* 2010). Its expression pattern in ciliated tissues suggested it may be involved in ciliogenesis (McClintock *et al.* 2008), but our data are the first to demonstrate that it has a role in ciliogenesis and affects cilia length and cell-cycle progression. NXN is a nucleoredoxin that inhibits Wnt/β-catenin signaling (Funato *et al.* 2006) and inhibits planar cell polarity (Funato *et al.* 2008). Disruption of Wnt signaling may affect cilia so it is possible that ciliary phenotypes seen in NXN knockdown cells are the result of a defect in this pathway. Unlike other Wnt pathway mutants however, NXN also leads to disengagement of the basal bodies/centrioles and may play a novel role or multiple roles in ciliogenesis. GLOD4 contains a glyoxalase domain and thus may function as an enzyme. It was previously shown to be downregulated in a canine model of retinitis pigmentosa (Genini *et al.* 2010), but we are the first to show a ciliary and cell cycle phenotype after knockdown.

SPATA4 is up-regulated during spermatogenesis in several organisms (Liu *et al.* 2004a; Liu *et al.* 2005a; Liu *et al.* 2005b; Xie *et al.* 2007) although its role in spermatogenesis is not clear. From our data, SPATA4 knockdown had no effect on cilia length, but fewer cells were able to build a cilium. Knockdown of SPATA4 also leads to an arrest of cells in S-phase. Basal bodies must be released from cilia to become centrioles and initiate duplication in S phase. By examining cells that cannot progress through S-phase, we highlight the lack of cilia in S phase cells. It may be that SPATA4 is required for cell cycle progression during spermatogenesis.

All of our ciliary phenotypes were rescued by shRNA-resistant copies of our genes. The rescue constructs also had C-terminal YFP tags to allow for localization of the proteins. It is surprising that none of our genes localized to the basal bodies or cilia. ZMYND10 had the most distinctive localization of cytoplasmic puncta, which may suggest its involvement in ciliary protein trafficking like has been shown for other proteins (Follit *et al.* 2006; Knodler *et al.* 2010; Donaldson and Jackson 2011). NXN, GLOD4, and SPATA4 did not localize to any distinctive cellular structure. This lack of localization has been shown previously for NXN (Kurooka *et al.* 1997; Funato *et al.* 2006) and SPATA4 (Duarte *et al.* 2011). The shRNA resistant copies of these genes are functional because they rescue the ciliary phenotypes and these genes are clearly involved in ciliogenesis because upon knockdown the cells have fewer cilia and/or short cilia. It is possible that these proteins are found in cilia or basal bodies at a specific part of the cell cycle. It is also possible that they may be cytoplasmic assembly components like DNAAF3 (Mitchison *et al.* 2012). In a recent paper looking at proteomics in primary cilia (Ishikawa *et al.* 2012), the authors generated GFP and FLAG tagged constructs of 18 of identified hits and found only 8 that localize to primary cilia. The authors interpreted this such that 10 were not *bona fide* ciliary components, but our data suggest that more genes than previously thought that are involved in ciliogenesis, but do not localize to the cilium.

It has been known for three decades that cilia are generally found on cells that have exited the cell cycle but only more recently has it been appreciated that genes involved in ciliogenesis also have roles in cell-cycle progression. Our data add to the list of known genes involved in ciliogenesis and cell-cycle progression. It is not clear what signals control cilia absorption and reentry of the cell cycle. Cilium absorption requires HEF1, AuroraA, and HDAC6, although these genes appear to function independent of cell-cycle progression (Pugacheva *et al.* 2007). The interaction between HEF1 and AuroraA activates HDAC6 to deacetylate the acetylated microtubules in the cilium, which causes them to become unstable and allows resorption to occur (Pugacheva *et al.* 2007). NDE1 and TCTEX-1 are also required for the transition from G_0_ to mitosis and their depletion causes cell cycle arrest (Kim *et al.* 2011; Li *et al.* 2011). Depletion of NDE1 arrests cells leading to longer cilia (Kim *et al.* 2011). Furthermore, it was found that cells express low levels of NDE1 in G_0_ where it localizes to the mother centriole, but greater levels in mitosis. Overexpression of NDE1 results in shorter cilia, which suggests that NDE1 regulates ciliary length (Kim *et al.* 2011). Like, NDE1, TCTEX-1, a dynein light chain, is also needed for cell cycle re-entry in a cilia-dependent manner (Li *et al.* 2011) although there is some discrepancy as to whether it also regulates ciliary length (Li *et al.* 2011; Palmer *et al.* 2011).

Another gene involved in ciliary absorption is the gene *PIFO* that is expressed in the mouse embryonic node (Kinzel *et al.* 2010). Two human patients haploinsufficient for *PIFO* show cilia defects, centrosome overduplication, and mitotic defects stemming from uncoupling of cilia resorption and cell cycle progression. The basal body fails to detach from the cilium although centrosome duplication still occurs causing severe mitotic defects including both mitotic spindles and cilia in the same cells (Kinzel *et al.* 2010). Interestingly, *PIFO* appears to only be conserved in vertebrates, thus it may not be part of a conserved mechanism for cilia resorption. Our results show SPATA4 has similar phenotypes to *PIFO* mutant cells including centrosome over-duplication and cell-cycle arrest. Because we only examined knockdown phenotypes of genes that were conserved between Chlamydomonas and humans, GLOD4 and SPATA4 may be part of a conserved mechanism of cilia resorption and warrant further study.

Whole-genome transcriptome analysis gives us insight into entire pathways required for ciliogenesis. The cilia assembly pathway is tightly linked to the cell cycle in mammalian cells as only cells in G_0_/G_1_ have cilia. Most of the genes we tested have both cilia and cell cycle effects that support the idea that these two cellular processes are intimately involved. Further analysis of additional genes will help to provide more mechanistic understanding the coordination of these pathways.

## Supplementary Material

Supporting Information
