# Supplementary material for: Identification of Cilia Genes That Affect Cell-Cycle Progression Using Whole-Genome Transcriptome Analysis in Chlamydomonas reinhardtti
Source: G3 (Bethesda). 2013 Jun 1;3(6):979–91. doi: 10.1534/g3.113.006338 (PMC3689809; doi:10.1534/g3.113.006338)
Supplement: Supporting Information [file supp_3_6_979__index.html]

Identification of Cilia Genes That Affect Cell-Cycle Progression Using Whole-Genome Transcriptome Analysis in Chlamydomonas reinhardtti — Supporting Information 

# Identification of Cilia Genes That Affect Cell-Cycle Progression Using Whole-Genome Transcriptome Analysis in *Chlamydomonas reinhardtti*

## Supporting Information for Albee *et al.*, 2013

**Files in this Data Supplement:**

- Supporting Information - Tables S1-S5 (PDF, 157 KB)
- Table S1 - shRNA sequences used for gene knockdown experiments (PDF, 74 KB)
- Table S2 - Primers used for qRT-PCR (PDF, 81 KB)
- Table S5 - List of genes involved in ergosterol biosynthesis (PDF, 68 KB)
- Table S3 - List of all genes upregulated at least 2.5 fold during ciliogenesis in *Chlamydomonas* (.xlsx, 366 KB)
- Table S4 - List of IFT genes, purified axonemal genes, central pair genes, and radial spoke genes that are found upregulated during ciliogenesis (.xlsx, 25 KB)
