# Supplementary material for: Identification of Cilia Genes That Affect Cell-Cycle Progression Using Whole-Genome Transcriptome Analysis in Chlamydomonas reinhardtti
Source: G3 (Bethesda). 2013 Jun 1;3(6):979–91. doi: 10.1534/g3.113.006338 (PMC3689809; doi:10.1534/g3.113.006338)
Supplement: Supporting Information [file supp_g3.113.006338_006338SI.pdf]

G3 Genes | Genomes | Genetics

Identification of cilia genes that affect cell cycle progression using whole genome transcriptome analysis in *Chlamydomonas reinhardtti*

Alison J. Albee*, Alan L. Kwan§[1], Huawen Lin*, David Granas §, Gary D. Stormo §, and Susan K. Dutcher*

- *Department of Genetics, Washington University School of Medicine, St. Louis, MO, USA
- §Center for Genomic Sciences and System Biology Washington University, St. Louis, MO USA

[1] current address: Molecular and Cellular Technologies, Platform Technology and Science GlaxoSmithKline Research and Development, Collegeville, PA USA

DOI: 10.1534/g3.113.006338

Table S1 shRNA sequences used for gene knockdown experiments

	Gene	Abbreviation	shRNA Sequence
*	control		ACTACACCATCGTGGAACAGT
	GLOD4	GL-16	CGACAGAACATCATGTGAGAT
	GLOD4	GL-90	GAGTGGTTTGCCAAACACAAT
	GLOD4	GL-82	GATGAAGCATTTCGAGAACTT
*	GLOD4	GL-30	GCAATGACTTTATGGGAATCA
	NXN	NX-73	CCTGGTGGAATCCTACCGGAA
	NXN	NX-68	GCGTCTATTTCTCCGCACATT
	NXN	NX-48	CCAACATTCCATCACTAATAT
	NXN	NX-13	GAATGACTTCCTAGCAGAGAA
*	NXN	NX-45	GCTCAAACTTTGGAACAAATA
	SPATA4	SP-10	CACCATCTCTAGCCATAATAA
	SPATA4	SP-72	CAATGAACTTAAAGCGGAGTT
	SPATA4	SP-79	CCAGAATGGTTTGATGTGAAA
*	SPATA4	SP-96	CAAGCTGGACAACATTCTTAT
	UPF1	UP-36	GCATCTTATTCTGGGTAATAA
	UPF1	UP-53	GCTGAGTTGAACTTCGAGGAA
	UPF1	UP-24	CCAACCCGATAAACCGATGTT
	UPF1	UP-28	GCAAGGTATGGCGTCATCATT
*	UPF1	UP-11	GCCTACCAGTACCAGAACATA
	ZMYND10	ZY-76	GAAGCTGAACATGCAAGCCAT
	ZMYND10	ZY-54	GATGCCAGAATGAGTGGTATT
	ZMYND10	ZY-26	GTCTTGGACTTGGTAGACTAT
*	ZMYND10	ZY-13	CCGTATGCTTAGCACACACAA

Table S2 Primers used for qRT-PCR

Gene	Forward Primer 5'→3'	Reverse Primer 5'→3
ABCA	CTCTGTACGCCACCAGACG	GGTTCAGCAGGAGCTTGTTC
ACTA	GCACAGAGCCTCGCCTTTGCC	CATGCCCACCATCACGCCCTGG
FAP178	CGAGACAGGCTACAACGACA	TTGAGAAGATTTCCGCCACT
GAPDH	CCTGTTCGACAGTCAGCCGCAT	CAGGCGCCCAATACGACCAA
GLOD4	GGCGCGTTTCTATCGGGACGT	GCGTTGCTGACAGCCTGGCTA
GLOD4 Chlamydomonas	CACATCCATGCCGTAACTTG	CAAGCTGGTACGACTTGACG
KCN1	CAGATAGCGGACAACATCCA	GTCCGACTCAGTCATCAGCA
KLP1	AGCGTATCGGTGAATGTTCC	GCCGTACGCGAAGATAGTTC
MOT8	CCGAATACCTCAGGCTGTTG	GTCTTCATGCTGCTGGCTAC
NXN	CGGTAACTGTGCCCCTTCACCG	GTTTGAGCGTTGCTGGGTTCCG
NXN Chlamydomonas	AGCCCAAGGTCTTCCAGATT	AAACACCACCTCCCAGTCAG
ODA6	GCCAAGTTCAACCTCAAGGA	TGTTCTCCACGCACTCATTC
RSP3	GCATCGACGAAGAAGAGGAC	AGGTCACCGTTCTCGATCTG
SPATA4	GGCACAGTTGGAGAAGTTCCTGGC	CCTGGAAACCAGGGGTAAACGCA
TUA1	CCTTCTCCTTCCCCTGATTT	GAAGGTGTTGAAGGCATCGT
UPF1	CCCGGGAGGCCATCATCCCA	AGCAGGCCCGTTGGCTTGTC
ZMYND10	CTTGGACTTGGTAGACTATTGC	ATCTCAAATTCCATCAGCTCT
ZMYND10 Chlamydomonas	GTCGCTGTCAGAGGACTTCG	AGCTTGGTCATAGCGAGCAT

Tables S3-S4 are available for download at http://www.g3journal.org/lookup/suppl/doi:10.1534/g3.113.006338/-/DC1.

Table S3 List of all genes upregulated at least 2.5 fold during ciliogenesis in *Chlamydomonas*

Table S4 List of IFT genes, purified axonemal genes, central pair genes, and radial spoke genes that are found upregulated during ciliogenesis

Table S5 List of genes involved in ergosterol biosynthesis

Enzyme Name	Fungal gene	Chlamydomonas gene name	Maximal change during regeneration
**Mevalonate independent 2-C-methyl-D-erythritol 4-phosphate (MEP) pathway**			
DXS		g356350	+1.1x
IspC/IspE/DXR		g546050	+1.6x
IspD		g16770	-2.0x
IspF		g503550	-1.3x
IspG/HDS1		g490350	-1.35
IspH		g372950	-1.1x
IDI/IDH1		g11474	+2.0x
**Isoprenoid biosynthesis pathway from delta-3-isopentenyl-pyrophosphate**			
Farnesyl pyrophosphate synthetase	*ERG20*	g207700	+2.4x
Farnesyl diphosphate franesyl transferase	*ERG9*	g175250	+1.4x
Squalene mono-oxygenase	*ERG1*	g17770	+2.3x
Lanosterol synthase	*ERG7*	g011100	+3.5x
Lanosterol 14-alpha demethylase	*ERG11*	g092350	+16.3x
C-14 sterol reductase	*ERG24/4*	g076800	+4.4x
C-4 methyl sterol oxidase sterol desaturase	*ERG25*	g103500	+2.1x
C3-sterol dehydrogenase	*ERG26*	g518650	+1.5x
Delta 24 sterol C methyltransferase	*ERG6*	g500500	+7.2x
C5 sterol desaturase	*ERG3*	g663950	+7.9x
C-22 sterol desaturase	*ERG5*	g11457	+6.5x
