# Supplementary material for: Identification of Cilia Genes That Affect Cell-Cycle Progression Using Whole-Genome Transcriptome Analysis in Chlamydomonas reinhardtti
Source: G3 (Bethesda). 2013 Jun 1;3(6):979–91. doi: 10.1534/g3.113.006338 (PMC3689809; doi:10.1534/g3.113.006338)
Supplement: Supporting Information [file supp_g3.113.006338_TableS1.pdf]

Table S1   shRNA sequences used for gene knockdown experiments

	Gene	Abbreviation	shRNA Sequence
*	control		ACTACACCATCGTGGAACAGT
	GLOD4	GL-16	CGACAGAACATCATGTGAGAT
	GLOD4	GL-90	GAGTGGTTTGCCAAACACAAT
	GLOD4	GL-82	GATGAAGCATTTCGAGAACTT
*	GLOD4	GL-30	GCAATGACTTTATGGGAATCA
	NXN	NX-73	CCTGGTGGAATCCTACCGGAA
	NXN	NX-68	GCGTCTATTTCTCCGCACATT
	NXN	NX-48	CCAACATTCCATCACTAATAT
	NXN	NX-13	GAATGACTTCCTAGCAGAGAA
*	NXN	NX-45	GCTCAAACTTTGGAACAAATA
	SPATA4	SP-10	CACCATCTCTAGCCATAATAA
	SPATA4	SP-72	CAATGAACTTAAAGCGGAGTT
	SPATA4	SP-79	CCAGAATGGTTTGATGTGAAA
*	SPATA4	SP-96	CAAGCTGGACAACATTCTTAT
	UPF1	UP-36	GCATCTTATTCTGGGTAATAA
	UPF1	UP-53	GCTGAGTTGAACTTCGAGGAA
	UPF1	UP-24	CCAACCCGATAAACCGATGTT
	UPF1	UP-28	GCAAGGTATGGCGTCATCATT
*	UPF1	UP-11	GCCTACCAGTACCAGAACATA
	ZMYND10	ZY-76	GAAGCTGAACATGCAAGCCAT
	ZMYND10	ZY-54	GATGCCAGAATGAGTGGTATT
	ZMYND10	ZY-26	GTCTTGGACTTGGTAGACTAT
*	ZMYND10	ZY-13	CCGTATGCTTAGCACACACAA
