# Supplementary material for: Identification of Cilia Genes That Affect Cell-Cycle Progression Using Whole-Genome Transcriptome Analysis in Chlamydomonas reinhardtti
Source: G3 (Bethesda). 2013 Jun 1;3(6):979–91. doi: 10.1534/g3.113.006338 (PMC3689809; doi:10.1534/g3.113.006338)
Supplement: Supporting Information [file supp_g3.113.006338_TableS2.pdf]

Table S2 Primers used for qRT-PCR

Gene	Forward Primer 5'→3'	Reverse Primer 5'→3
ABCA	CTCTGTACGCCACCAGACG	GGTTCAGCAGGAGCTTGTTC
ACTA	GCACAGAGCCTCGCCTTTGCC	CATGCCCACCATCACGCCCTGG
FAP178	CGAGACAGGCTACAACGACA	TTGAGAAGATTTCCGCCACT
GAPDH	CCTGTTCGACAGTCAGCCGCAT	CAGGCGCCCAATACGACCAA
GLOD4	GGCGCGTTTCTATCGGGACGT	GCGTTGCTGACAGCCTGGCTA
GLOD4 Chlamydomonas	CACATCCATGCCGTAACTTG	CAAGCTGGTACGACTTGACG
KCN1	CAGATAGCGGACAACATCCA	GTCCGACTCAGTCATCAGCA
KLP1	AGCGTATCGGTGAATGTTCC	GCCGTACGCGAAGATAGTTC
MOT8	CCGAATACCTCAGGCTGTTG	GTCTTCATGCTGCTGGCTAC
NXN	CGGTAACTGTGCCCCTTCACCG	GTTTGAGCGTTGCTGGGTTCCG
NXN Chlamydomonas	AGCCCAAGGTCTTCCAGATT	AAACACCACCTCCCAGTCAG
ODA6	GCCAAGTTCAACCTCAAGGA	TGTTCTCCACGCACTCATTC
RSP3	GCATCGACGAAGAAGAGGAC	AGGTCACCGTTCTCGATCTG
SPATA4	GGCACAGTTGGAGAAGTTCCTGGC	CCTGGAAACCAGGGGTAAACGCA
TUA1	CCTTCTCCTTCCCCTGATTT	GAAGGTGTTGAAGGCATCGT
UPF1	CCCGGGAGGCCATCATCCCA	AGCAGGCCCGTTGGCTTGTC
ZMYND10	CTTGGACTTGGTAGACTATTGC	ATCTCAAATTCCATCAGCTCT
ZMYND10 Chlamydomonas	GTCGCTGTCAGAGGACTTCG	AGCTTGGTCATAGCGAGCAT
