# Supplementary material for: Identification of Cilia Genes That Affect Cell-Cycle Progression Using Whole-Genome Transcriptome Analysis in Chlamydomonas reinhardtti
Source: G3 (Bethesda). 2013 Jun 1;3(6):979–91. doi: 10.1534/g3.113.006338 (PMC3689809; doi:10.1534/g3.113.006338)
Supplement: Supporting Information [file supp_g3.113.006338_TableS5.pdf]

Table S5 List of genes involved in ergosterol biosynthesis

Enzyme Name	Fungal gene	Chlamydomonas gene name	Maximal change during regeneration
**Mevalonate independent 2-C-methyl-D-erythritol 4-phosphate (MEP) pathway**			
DXS		g356350	+1.1x
IspC/IspE/DXR		g546050	+1.6x
IspD		g16770	-2.0x
IspF		g503550	-1.3x
IspG/HDS1		g490350	-1.35
IspH		g372950	-1.1x
IDI/IDH1		g11474	+2.0x
**Isoprenoid biosynthesis pathway from delta-3-isopentenyl-pyrophosphate**			
Farnesyl pyrophosphate synthetase	*ERG20*	g207700	+2.4x
Farnesyl diphosphate franesyl transferase	*ERG9*	g175250	+1.4x
Squalene mono-oxygenase	*ERG1*	g17770	+2.3x
Lanosterol synthase	*ERG7*	g011100	+3.5x
Lanosterol 14-alpha demethylase	*ERG11*	g092350	+16.3x
C-14 sterol reductase	*ERG24/4*	g076800	+4.4x
C-4 methyl sterol oxidase sterol desaturase	*ERG25*	g103500	+2.1x
C3-sterol dehydrogenase	*ERG26*	g518650	+1.5x
Delta 24 sterol C methyltransferase	*ERG6*	g500500	+7.2x
C5 sterol desaturase	*ERG3*	g663950	+7.9x
C-22 sterol desaturase	*ERG5*	g11457	+6.5x
